# ggpubfigs: Colorblind-Friendly Color Palettes and ggplot2 Graphic System Extensions for Publication-Quality Scientific Figures

**DOI:** 10.1128/MRA.00871-21

**Published:** 2021-11-04

**Authors:** Jacob L. Steenwyk, Antonis Rokas

**Affiliations:** a Department of Biological Sciences, Vanderbilt University, Nashville, Tennessee, USA; Indiana University, Bloomington

## Abstract

Clear and effective figures are central to successfully communicating scientific data. Here, we present ggpubfigs, an R package with colorblind-friendly color palettes and extensions of the ggplot2 graphic system, which helps make publication-quality scientific figures from quantitative data; ggpubfigs is an open-source and user-friendly tool that is available from https://github.com/JLSteenwyk/ggpubfigs.

## ANNOUNCEMENT

Scientific figures are graphical representations of scientific data (1). Several tools have been developed to generate scientific figures in numerous computer-programming languages, including seaborn (2) and Matplotlib (3) in the Python programming language and Lattice (4) and ggplot2 (5) in the R programming language. These and other data visualization software programs have empowered researchers with the ability to generate scientific figures from diverse sources of quantitative data. As a result, methods and standards for effective scientific figures, which we define as accurate, clear, and precise representations of scientific data, have been topics of rigorous debate, which is influenced in part by field, audience, and data type (6–8).

Although certain rules for effective scientific figures are context dependent and subject to change, some rules are broadly applicable to several disciplines, including in microbiology and the life sciences; these include two rules from the report by Rougier et al., i.e., “do not trust the defaults” and “use color effectively” (1). For the first rule, the authors suggest that default plotting parameters (e.g., font sizes and tick marks) are sufficient to make a scientific figure but insufficient to make the best scientific figure. For the second rule, the authors suggest that color is an important component of human vision and thus is equally important in making scientific figures. Effective color use can also make scientific figures more accessible. For example, 8% and 0.4% of European Caucasian men and women, respectively, are red-green color deficient (9). Therefore, effective figure making is also a matter of inclusion.

**FIG 1:**
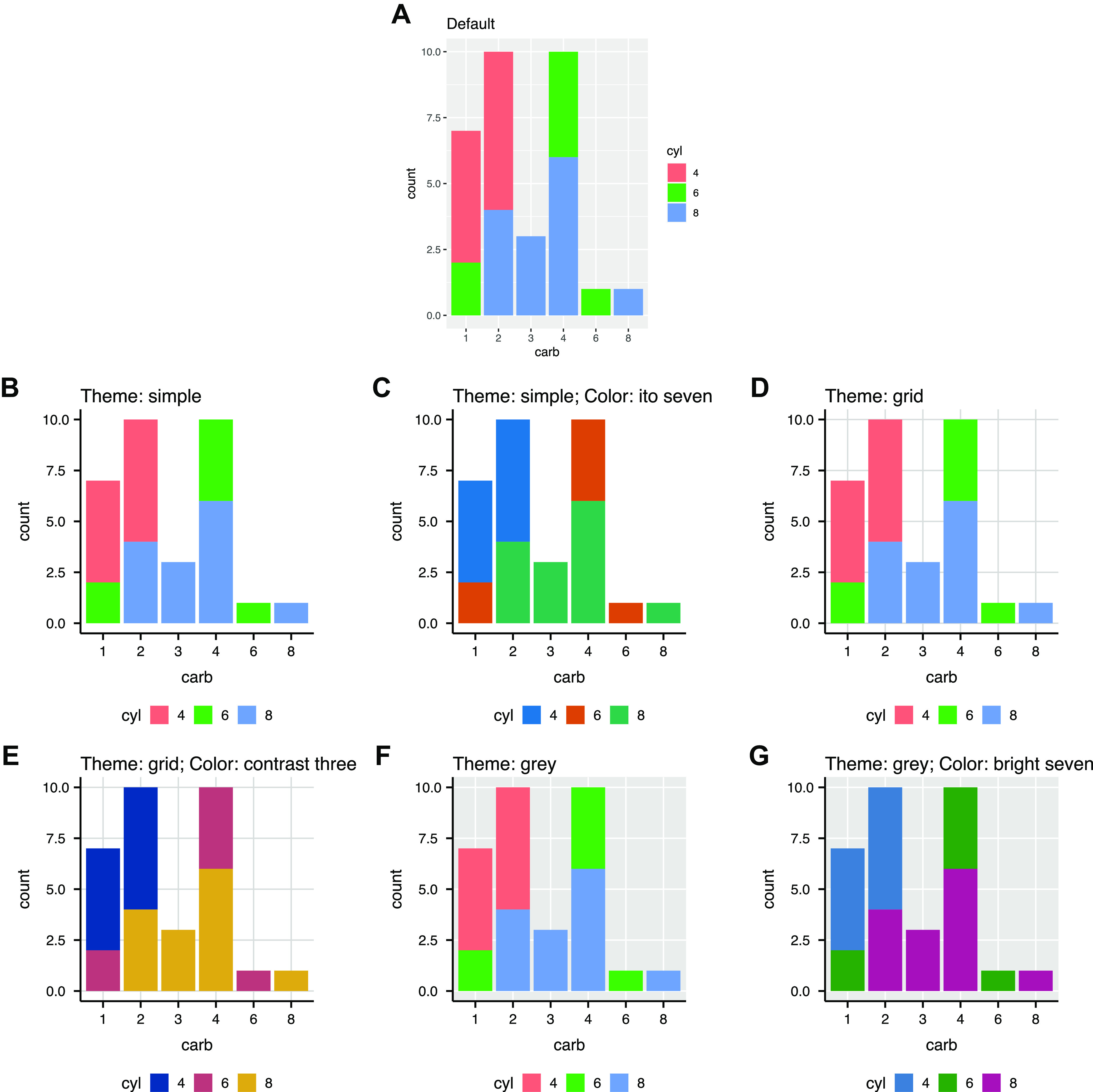
Examples of ggplot2 extensions and color palettes available in ggpubfigs. (A) Default scientific visualization made using ggplot2. (B to G) Modified scientific figures made using the simple theme (B), the simple theme with the ito seven color palette (C), the grid theme (D), the grid theme with the contrast three color palette (E), the gray theme (F), and the gray theme with the bright seven color palette (G). Data are from the mtcars data frame available through the data sets package.

To facilitate effective figure making, we present ggpubfigs, an R package with colorblind-friendly color palettes and ggplot2 extensions that facilitate the generation of publication-quality scientific figures for quantitative data (https://github.com/JLSteenwyk/ggpubfigs). More specifically, ggpubfigs contains six color palettes that are colorblind friendly and aim to increase the accessibility of scientific figures and eight themes that modify 21 parameters of a default ggplot2 figure. To demonstrate how ggpubfigs can improve scientific figures in R, we compare the default ggplot2 settings (Fig. 1A) with those modified using extensions or colorblind-friendly color palettes available in ggpubfigs (Fig. 1B to G). Users can create additional modifications to a scientific figure according to their specific needs.

Color palettes can be accessed using the friendly_pal() function. For example, friendly_pal(“contrast_three”) will provide users access to an object of class palette that contains the hex codes for the contrast three color palette. Color palettes can be converted into a colormap of *N* colors (which may be useful for plotting data as a heatmap) using the following command: friendly_pal(“contrast_three”, *N*, type = “continuous”). Themes that modify ggplot2 plots can be appended to the end of ggplot2 plotting commands. For example, to use the simple theme in ggpubfigs, theme_simple(), an object of class gg theme, should be appended to the end of a ggplot2 plotting command.

We anticipate that ggpubfigs will assist researchers in generating effective scientific figures that are accessible to broad audiences, including those that are colorblind.

### Data availability.

The ggpubfigs package is freely available under the MIT license and is available for download at GitHub (https://github.com/JLSteenwyk/ggpubfigs). The GitHub repository comes complete with installation instructions and tutorials. Installing ggpubfigs is simple and requires executing only one command. Tutorials detail how to use color palettes for qualitative data and continuous and discrete quantitative data, as well as utilizing ggplot2 theme extensions.
